# Identification of soil characteristics using Vp & Vs tomograms for hazard assessment: case study from Ain Sokhna area, Egypt

**DOI:** 10.1038/s41598-026-63329-x

**Published:** 2026-08-11

**Authors:** Ahmed El-khteeb, Walid M. Mabrouk, Muhammad A. El Hameedy, Ahmed M. H. Metwally

**Affiliations:** Geophysics Department, Faculty of Science, Cairo University, Giza, 12613 Egypt

**Keywords:** Ain Sokhna, Seismic refraction, Hazard assessment, Geotechnical, NEHRP, Engineering, Environmental sciences, Natural hazards, Solid Earth sciences

## Abstract

This study presents a detailed geotechnical characterization of an industrial development site in Ain Sokhna, Egypt, a strategic location within the new industrial zone of the Suez Canal, using integrated P-wave (V_p_) and S-wave (V_s_) tomography. The seismic velocity models reveal three subsurface layers, starting from unconsolidated wadi sediments at the surface to compacted sandstone bedrock at depth. Compressional wave V_p_ values ranging from 400 m/s in the near surface to 3500 m/s in the deepest layer, while shear wave V_s_ values rise from as low as 130 m/s to approximately 1896 m/s, reflecting a marked enhancement in material stiffness with depth. From these velocity profiles and estimated densities, the dynamic elastic moduli were derived. The shear modulus (G) increases from about 0.069 up to 7.91 GPa, and Young’s modulus (E) from 0.043 to 14.2 GPa, attended by a rise in bulk modulus (K) from the order of 0.01 to 10 GPa with depth. These high moduli values in the bedrock layer indicate a highly stiff, well-consolidated ground. Geotechnical parameters including stress ratio, material index, concentration index, standard penetration resistance (N-value), ultimate bearing capacity, and settlement were also calculated to measure soil and rock competence. The results indicate an increase in bearing capacity and decreasing settlement in deeper layers, consistent with the transition from loose soil to strong bedrock. Notably, the time-averaged V_s_ to 30 m depth (Vs₃₀) is consistently high across the site (800–1100 m/s), leading to a NEHRP Site Classification of B (rock/very stiff soil). This integrated geophysical-geotechnical approach provides a comprehensive assessment of subsurface conditions, yielding critical parameters for seismic hazard evaluation and safe foundation design in the Ain Sokhna region on the coastal side of the Gulf of Suez (GOS).

## Introduction

 The success of civil engineering projects is strongly dependent on a thorough understanding of subsurface conditions and the geotechnical properties of soils and rocks. Subsurface characterization plays a central role in foundation design, ensuring structural stability, and assessing seismic hazards^1,2^. Inadequate knowledge of geological and geotechnical settings can lead to major construction challenges, project delays, or even structural failure^3^. Conventional techniques such as trenching and boreholes, while providing valuable point-specific information, are limited in spatial coverage and often fail to capture the lateral and vertical heterogeneity inherent in complex geological environments. This limitation highlights the importance of geophysical methods, which offer non-destructive, cost-effective, and efficient means of obtaining continuous subsurface data across wide areas^4,5^.

Among the various geophysical approaches, seismic methods have proven especially powerful in site investigations and engineering applications. The seismic refraction method, in particular, has long been established as a standard tool for mapping bedrock depth and determining near-surface velocity structures^6–10^. With advances in data acquisition and inversion algorithms, Shallow Seismic Refraction Tomography (SSRT) has emerged as a superior approach to traditional refraction surveys, capable of resolving subtle lateral and vertical velocity variations and accurately imaging complex subsurface geometries. P-wave (Vp) tomography, which focuses on compressional wave velocities, is widely employed to delineate bedrock topography, evaluate the competency of foundation materials, and provide essential input for civil and geotechnical designs^11–13^.

Complementary to SRT is the Multichannel Analysis of Surface Waves (MASW), a seismic technique designed to extract shear-wave (Vs) velocity profiles from surface wave dispersion. MASW is particularly valuable because shear-wave velocities are directly related to the stiffness and dynamic behavior of soils, making them indispensable for the assessment of deformation characteristics and seismic response^14,15^. By overcoming limitations associated with P-wave methods such as difficulties in imaging velocity inversions MASW adds a crucial dimension to subsurface studies. The integration of both P-wave and S-wave seismic data enables a comprehensive understanding of lithological variations, saturation conditions, and elastic properties, thereby facilitating more reliable geotechnical and hazard assessments^16–18^.

The use of seismic velocity data extends beyond imaging and interpretation, as it provides a foundation for deriving a wide range of dynamic geotechnical and elastic parameters. Properties such as shear modulus, bulk modulus, Young’s modulus, Poisson’s ratio, and concentration index can be calculated directly from velocity measurements^19,20^. These parameters are essential for quantifying the strength, stiffness, and deformation capacity of subsurface materials, offering practical insights into both engineering design and seismic hazard evaluation. The accurate estimation of these properties is particularly critical for shallow sedimentary layers, where construction activity is often concentrated, and where heterogeneity in soil and rock units can significantly influence structural stability^21,22^.

In modern site investigations, geophysical techniques are increasingly employed to derive dynamic geotechnical parameters that are essential for both structural design and seismic performance evaluation. A particularly important parameter is the average shear-wave velocity to a depth of 30 m (Vs_30_), which has become the standard metric for classifying sites according to seismic response criteria^23,24^. Vs₃₀ plays a central role in assessing the suitability of a site for safe and resilient construction, as it directly informs the seismic site class defined by the National Earthquake Hazards Reduction Program (NEHRP). Beyond its application to hazard classification, detailed assessment of the geotechnical behavior of bedrock including subsurface layering, lithological composition, and elastic properties provides the geological and engineering input needed for the safe design of major infrastructure and critical facilities. In this context, Vs_30_ serves not only as a diagnostic measure of shallow subsurface stiffness but also as a key parameter linking geophysical observations to engineering practice^25,26^.

For the Ain Sokhna region, which is characterized by complex fault-controlled structures and hosts intensive industrial development, accurate estimation of Vs_30_ is especially critical^27^. Determining site classes and associated dynamic properties in such tectonically active settings ensure that foundation designs account for potential amplification effects and that infrastructure is constructed to withstand both operational loads and seismic hazards.

**Fig. 1:**
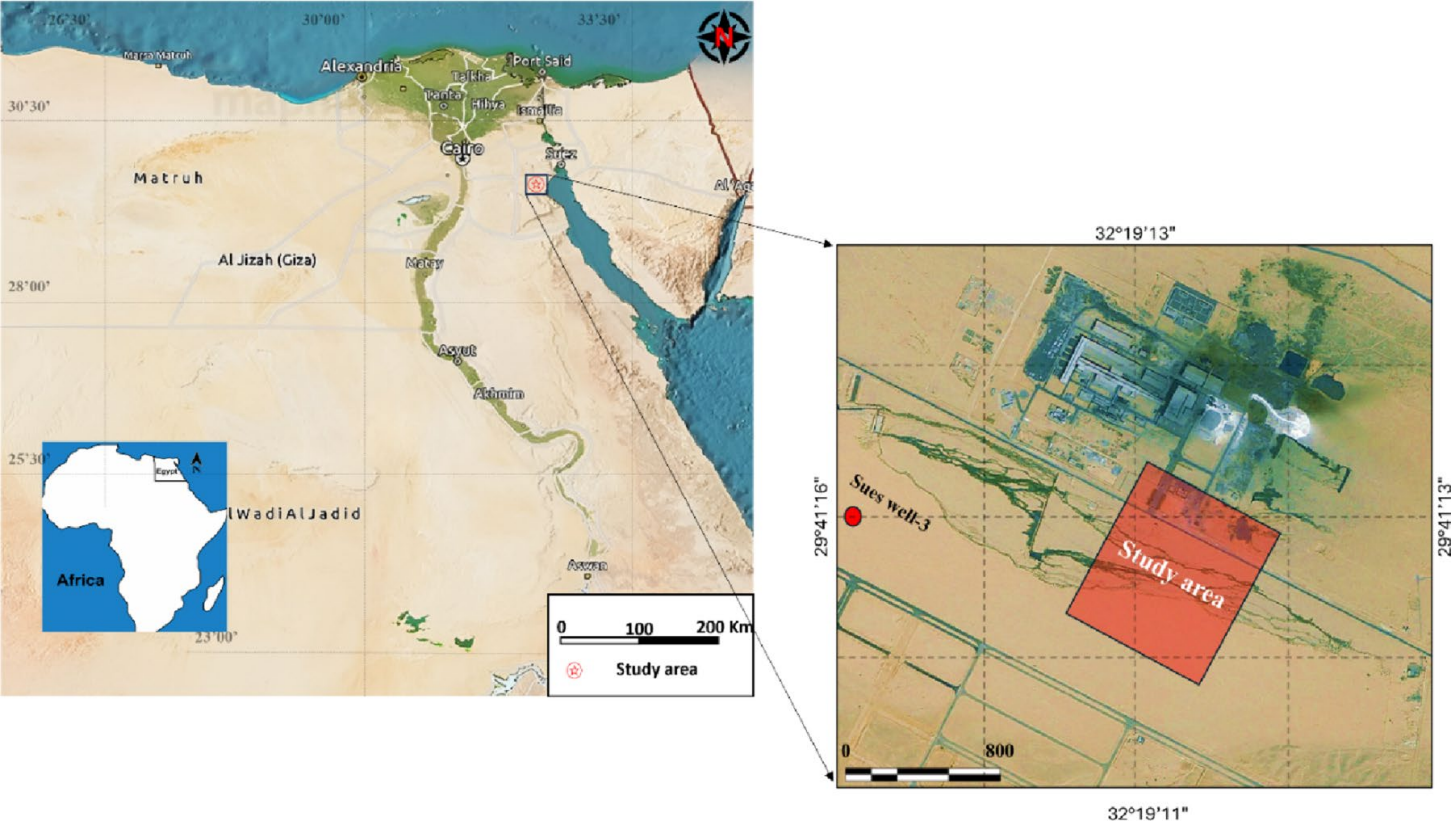
Location map of the study area.

The Ain Sokhna area, located on the western shore of the Gulf of Suez Egypt (Fig. 1), represents a strategic industrial zone currently undergoing extensive development. The region is of national importance due to its proximity to the newly established Suez port and its role in industrial and infrastructural expansion^28–30^. From a geological perspective, Ain Sokhna is characterized by Quaternary clastic deposits, Oligocene to Miocene rocks, and an underlying Eocene limestone plateau. Structurally, the area is strongly influenced by NW-SE and NE-SW trending normal faults associated with the Red Sea rifting system. This tectonic complexity, combined with lithological diversity, necessitates careful subsurface investigation to ensure safe foundation design and mitigate potential seismic hazards^31–33^.

Previous geophysical studies in Ain Sokhna have primarily utilized P-wave seismic refraction to map bedrock depth and identify major seismic layers. For example, El Hameedy et al. 2023^30^. delineated three principal geoseismic layers unconsolidated Wadi sediments, consolidated Wadi sediments, and fractured to intact sandstone bedrock while highlighting the role of fault-controlled bedrock topography. These contributions have advanced our understanding of the region; however, they remain largely focused on compressional-wave data, with limited integration of shear-wave information and MASW derived profiles. This leaves a notable gap in the comprehensive characterization of soil dynamic properties and elastic moduli, parameters that are critical for both hazard assessment and resilient engineering design.

## Data and methods

**Fig. 2:**
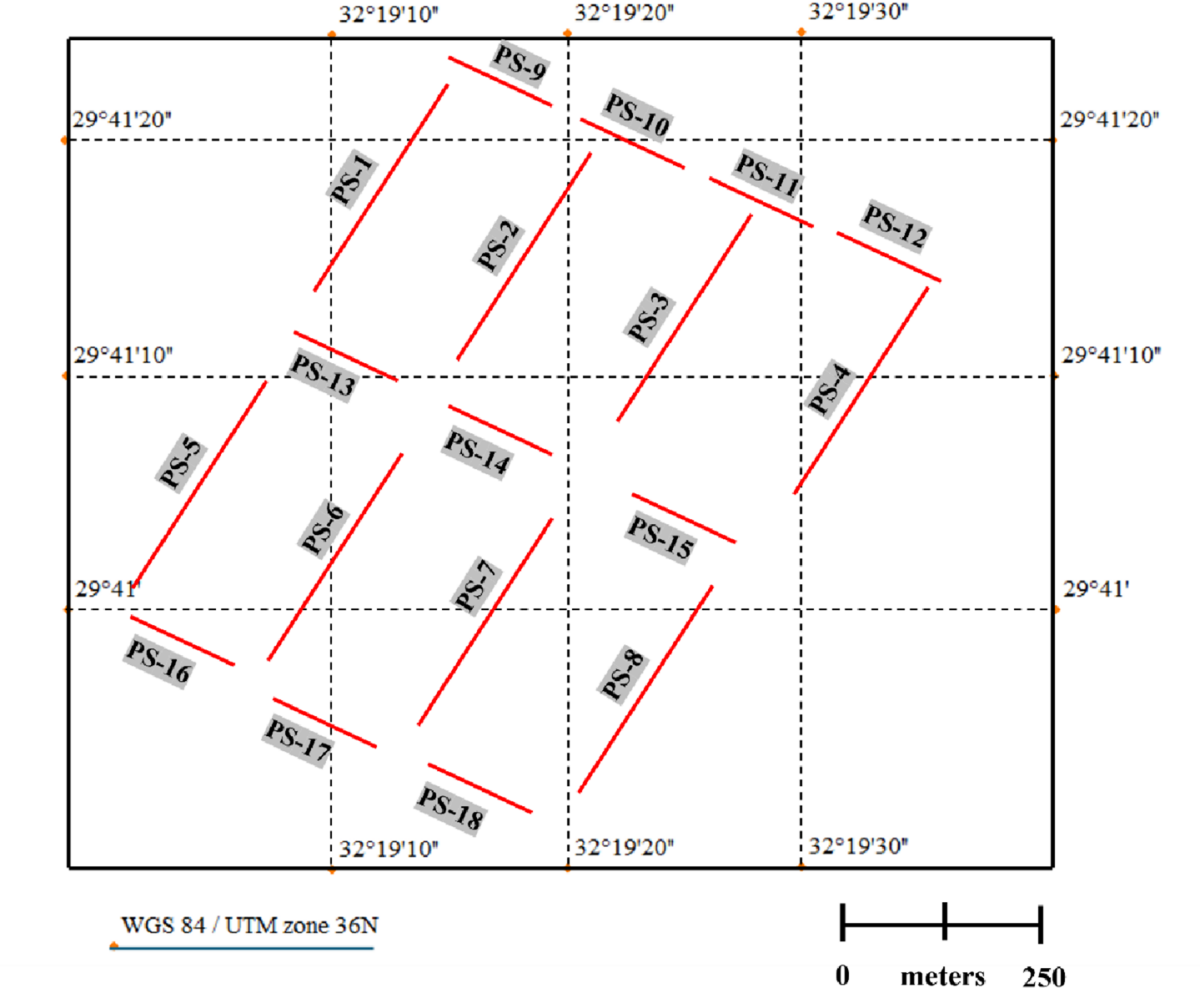
Layout of the acquired seismic profiles across the study area.

The field survey involved 18 seismic refraction profiles (Fig. 2) covering a total length of approximately 3150 m along the Cairo-Suez Road in the Ain Sokhna area. In addition, wireline logging data from the Suez well-3, drilled by the Egyptian Geological Survey and Mining Authority (EGSMA), were incorporated as a geological reference to enhance the reliability of interpretations. This combined methodology enabled the construction of detailed velocity tomograms, the estimation of key dynamic geotechnical properties, and the assessment of subsurface competency for engineering applications.

The objectives of this study are threefold:

1. To analyze compressional-wave (Vp) and shear-wave (Vs) velocities using SRT and MASW methods in order to delineate subsurface lithology and layering.
2. To calculate a comprehensive suite of dynamic geotechnical and elastic parameters for the identified soil and rock units.
3. To evaluate the competency of subsurface materials and assess their implications for hazard mitigation and future engineering developments in Ain Sokhna.
4. Calculating Vs30 to determine NEHRP site class.

### Geologic setting

The Ain Sokhna region is situated along the western margin of the Gulf of Suez within the Suez Governorate, Egypt. This area occupies a transitional zone between the coastal plain bordering the gulf and the elevated mountainous terrain of the Northern Galala and Gabal Ataqa ranges to the west. Owing to its strategic position, Ain Sokhna has become a focal point for industrial and infrastructural expansion, which necessitates detailed geotechnical and geophysical investigations^31^.

Geomorphologically, the study area can be divided into four principal units:

1. Coastal Plain:

This low-lying zone extends from the shoreline of the Gulf of Suez westward toward the highlands. It is predominantly covered by Quaternary clastic sediments, consisting of extensive sandy and gravelly deposits that form a gently eastward-dipping plain. These unconsolidated materials represent the youngest geomorphological features in the region.

2. Highlands:

The Northern Galala Plateau and Gabal Ataqa form the most prominent highland units. Gabal Ataqa exhibits steep escarpments, with elevations exceeding 900 m above sea level. The highlands are structurally controlled, reflecting the influence of major fault systems associated with the rifting of the Gulf of Suez.

3. Low-Elevation Hills:

Much of this unit is composed of sedimentary rocks spanning from the Upper Eocene through the Miocene. These include limestones, sandstones, and other clastic deposits, which form rolling hills and ridges between the highlands and the coastal plain.

4. Drainage Network:

The region hosts several significant drainage basins that transport sediments from the highlands to the Gulf of Suez. The most prominent are Wadi Hagoul, South Wadi Hagoul, Wadi Akheider Bada, and Wadi Ghweiba. These wadis strongly influence the sedimentary architecture of the area by supplying coarse-grained deposits to the coastal plain.

**Fig. 3:**
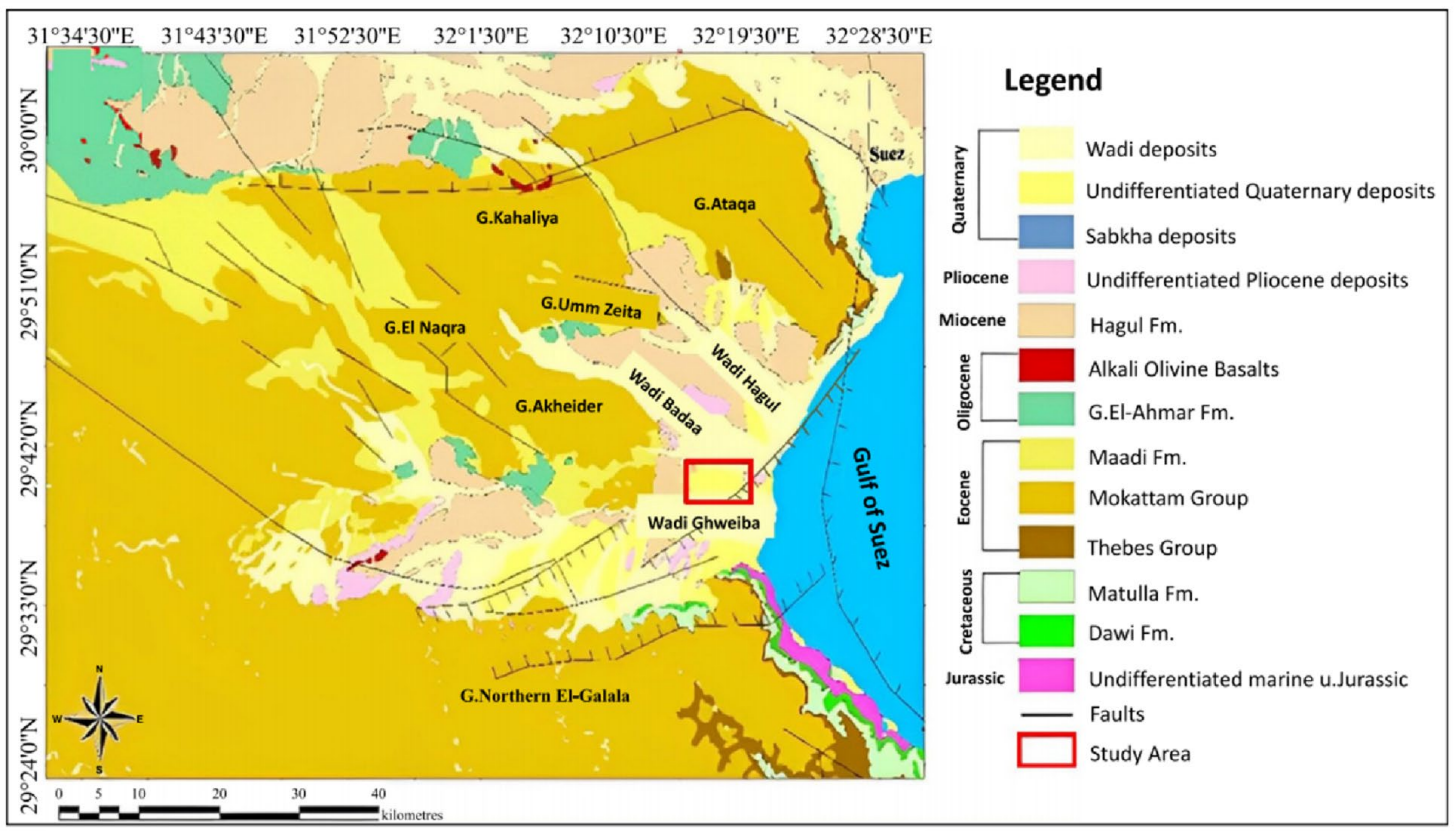
Geological map of the western side of Gulf of Suez after (Conco, 1987).

From a geological perspective (Fig. 3), the stratigraphy of Ain Sokhna records a long and complex history spanning the Eocene to Quaternary. The Eocene succession is represented by the Mokattam Formation (Middle Eocene) and the Maadi Formation (Upper Eocene). These are overlain unconformably by Oligocene deposits, which include both volcanic units (basaltic flows and dolerite intrusions) and sedimentary sequences such as sandstones and conglomerates. The Miocene succession is characterized by the Sadat Formation (Lower Miocene) and the Hagul Formation (Upper Miocene), both consisting mainly of sandstones and marls. Younger Pliocene deposits comprise gravels, sands, and flint pebbles, while the Quaternary sediments are dominated by unconsolidated alluvial and terrace deposits, including gravels, cobbles, boulders, and sands^27,28^.

Structurally, the region lies within the Gulf of Suez rift system and displays a distinctive extensional tectonic style. Numerous normal faults dissect the area, forming half-graben and domino-style fault blocks. Fault orientations are diverse but three major trends dominate: NNW-SSE, E-W, and WNW-ESE. The NW-SE faults are the most significant, controlling the development of major wadis such as Hagoul, Badaa, and Ghweiba. The E-W faults are thought to have initiated during the Late Eocene to Oligocene, while the WNW–ESE faults are prominent in the depression between Gabal Ataqa and the Northern Galala Plateau. These structural features collectively govern the subsurface topography, sediment distribution, and groundwater potential of the region^34,35^.

In summary, the Ain Sokhna area presents a complex geological and geomorphological framework shaped by interactions between sedimentary processes and extensional tectonics linked to the Red Sea and Gulf of Suez rifting. This combination of lithological variability and fault-controlled structures underscores the importance of integrating geophysical methods to accurately characterize the subsurface for engineering and hazard assessment purposes.

### Data acquisition

In order to characterize the subsurface conditions at Ain Sokhna, both compressional (P-wave) and shear (S-wave) seismic surveys were conducted using survey tomography and surface-wave recording. The seismic layout consisted of multiple profiles with lengths ranging between 120 and 240 m, oriented in both NE-SW and NW-SE directions to provide adequate coverage of the site. Geophones were deployed at 10 m intervals along each profile, employing spreads of 12 and 24 channels. For the P-wave survey, a 15 kg sledgehammer striking a steel plate served as the seismic source, while for the S-wave survey, horizontal strikes against a wooden plank were used to generate polarized shear waves suitable for MASW analysis.

Each spread was recorded using a Geometrix 24-channel seismograph (model 1125E) and a McSEIS-SX acquisition system, with multiple shot points positioned in forward, reverse, and offset geometries to ensure reliable first arrival picking and dispersion imaging. In total, 18 seismic lines were collected, producing an integrated survey length of approximately 3.15 km. Ten shot gathers per spread were acquired to maximize data redundancy and improve the signal-to-noise ratio (Table 1).

**Table 1:** Data acquisition parameters.

Parameter	*P*-wave Refraction Survey	S-wave & MASW Survey
Survey area	Ain Sokhna, Gulf of Suez, Egypt	Ain Sokhna, Gulf of Suez, Egypt
Number of spreads	28 spreads (Spreads 1–28)	28 spreads (Spreads 1–28)
Spread length	120 m and 240 m	120 m and 240 m
Recording system	24- and 12-channel digital seismographs	24- and 12-channel digital seismographs
Number of channels	12 or 24 geophones per spread	12 or 24 geophones per spread
Geophone type	10 Hz vertical geophones	10 Hz horizontal geophones
Geophone spacing (GS)	5 m	5 m
Shot spacing	10–20 m	10–20 m
Source type	15 kg sledgehammer on steel plate	15 kg sledgehammer against wooden plank
Acquisition directions	N–S and E–W oriented profiles	N–S and E–W oriented profiles
Shot geometry	Forward, reverse, off-end, and split	Forward, reverse, and split shots (East/West)
Total profiles	28 profiles (~ 3.15 km)	28 profiles (~ 3.15 km)

The P-wave dataset provided high-resolution first-arrival travel times for refraction tomography, while the S-wave survey was primarily used to extract dispersion curves and generate shear-wave velocity profiles through MASW inversion for each profile. Together, these complementary methods offered a robust dataset for imaging the velocity structure of the near surface, enabling accurate delineation of lithological boundaries and the derivation of key elastic parameters essential for geotechnical and seismic hazard assessment.

### Data processing

**Fig. 4:**
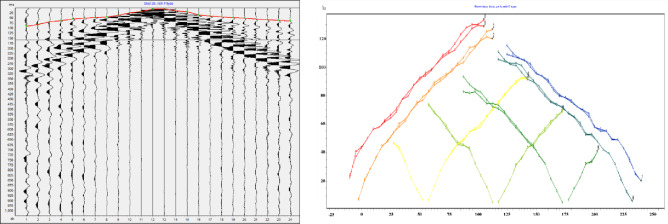
Examples of the picked seismic refraction data for Profile 1, illustrating the comparison between the observed and modeled travel time-distance curves derived from the survey.

**Fig. 5:**
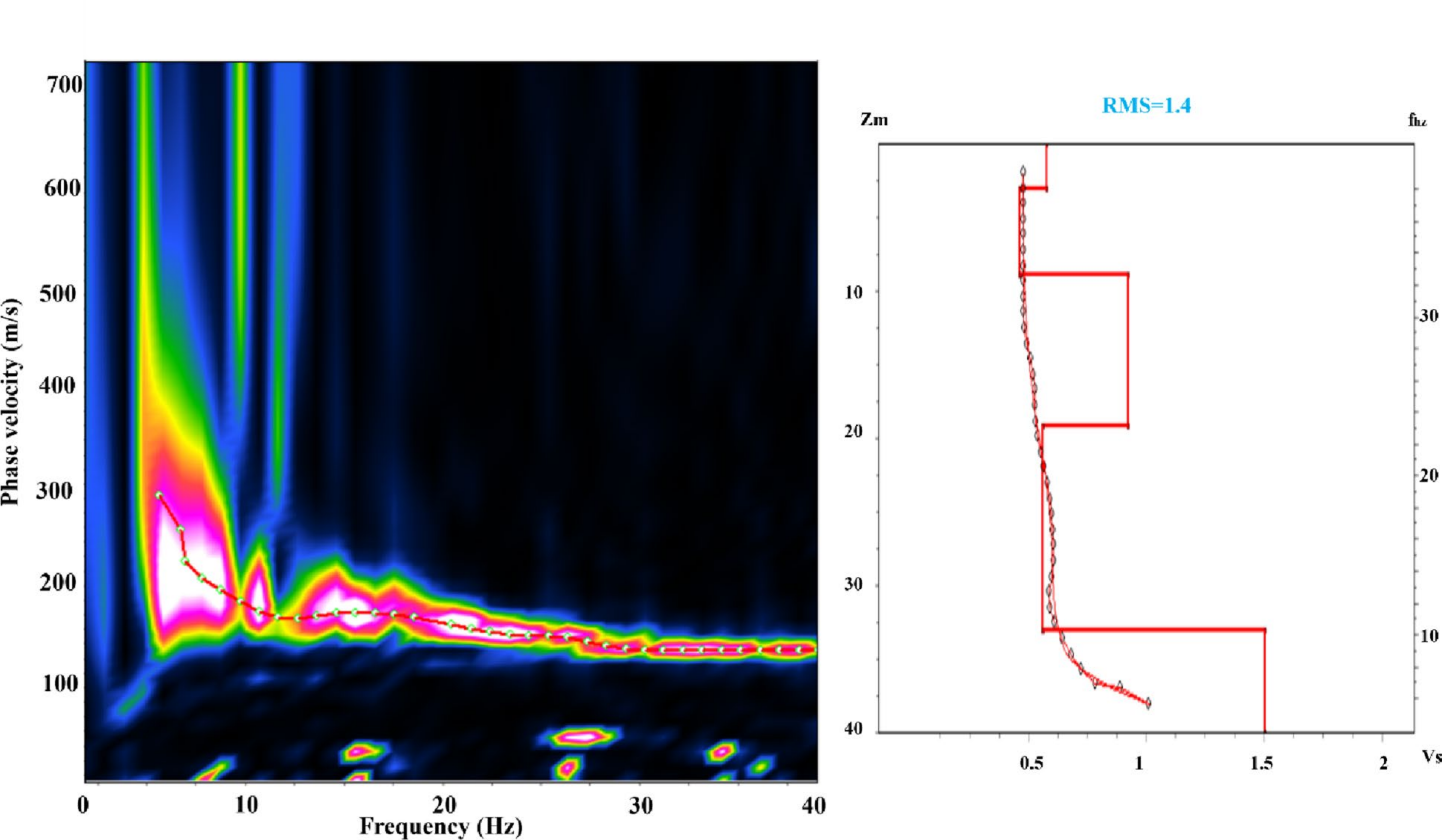
Generated dispersion curves for the analyzed profile, highlighting the fundamental-mode phase velocities used to construct the corresponding phase velocity curve.

All seismic data acquired in the Ain Sokhna survey were processed using the SeisImager/2D and Zond software, which incorporates comprehensive modules for refraction and tomography analysis based on delay-time and ray-tracing methods. For the P-wave records, the first arrivals were manually picked from each shot gather (Fig. 4). These arrivals were then used to construct time-distance curves after assigning the appropriate field geometry parameters, including spread length, geophone spacing, and source receiver offsets. Then the time-distance curves generated for each profile were analyzed. This step produced reliable seismic velocity distributions for compressional (Vp) and wave across the survey lines^36,37^. For the S-wave records the dispersion curves are generated and the phase velocities are picked to construct the phase velocity curve (Fig. 5).

P-wave velocity models were obtained through tomographic inversion of first-arrival travel times using a least-squares approach combined with ray-tracing forward modeling. A three-layer initial model was adopted, consisting of unconsolidated wadi deposits (Vp = 600 m/s), consolidated sediments (Vp = 1,100 m/s), and sandstone bedrock (Vp = 2,500 m/s). To improve model stability and reduce inversion artifacts, L2-norm regularization, velocity damping, and boundary smoothing were applied. The subsurface was discretized using a progressively refined block-based parameterization, and ray coverage was evaluated through hit-count analysis. The inversion converged after approximately nine iterations, yielding an average RMS travel-time misfit of 3.93 ± 0.58% across the eighteen profiles.

Shear-wave velocity (Vs) models were derived from MASW data acquired using a 15-kg sledgehammer source. Fundamental-mode dispersion curves were extracted using phase-shift analysis and inverted through a Gauss-Newton optimization scheme constrained by the corresponding P-wave models. Initial Vs estimates were guided by assumed Vp/Vs ratios of 1.90, 1.75, and 1.65 for the three identified layers. The inversion produced an average dispersion-curve RMS misfit of 6.36 ± 1.26%, with phase-velocity uncertainties of 28.8 ± 8.5 m/s.

The average depth of investigation reached approximately 40 m, while reliable characterization of the upper 30 m was achieved along the longer profiles, enabling robust Vs30 estimation. The resulting velocity models resolved three major subsurface units with distinct geophysical characteristics, in good agreement with lithological information from Suez Well-3.

**Fig. 6:**
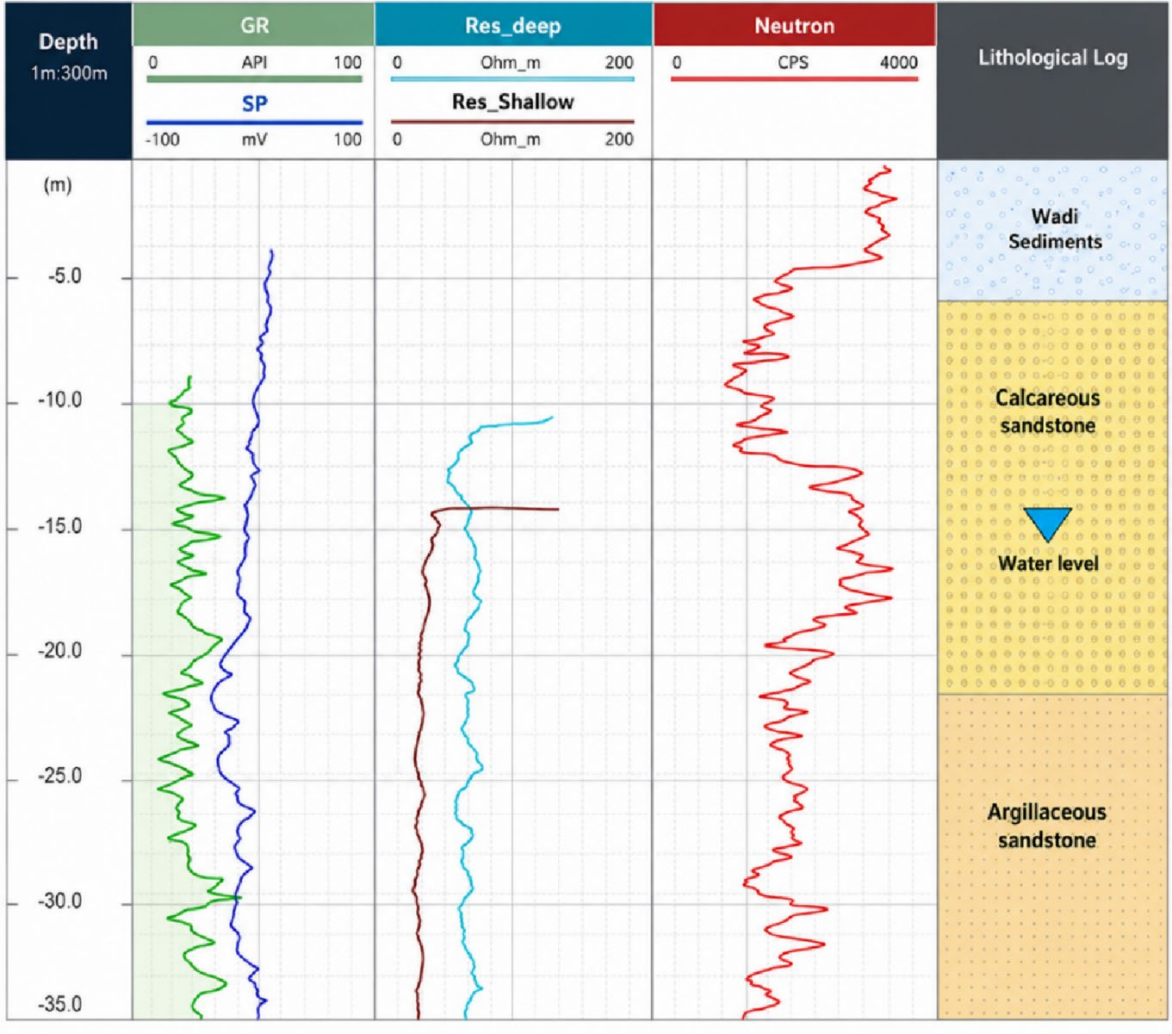
Wireline log data from the upper interval of Suez Well-3 located adjacent to the study area.

The final P-wave and S-wave velocity models were integrated into two-dimensional subsurface sections for each profile, providing a detailed characterization of lateral and vertical variations in seismic velocities. The P-wave tomograms delineated soil stratification and bedrock geometry, while the S-wave models resolved shear-wave velocity distributions essential for estimating elastic moduli and other dynamic geotechnical parameters. Hydrogeological conditions, particularly the regional groundwater table at approximately 14 m depth, were inherently incorporated into the velocity inversions and Gardner-derived density estimations, ensuring that the calculated elastic properties, including Poisson’s ratios (0.21–0.28 within the bedrock), accurately represent in-situ subsurface conditions. The integrated interpretation identified three principal subsurface units with distinct geophysical characteristics, in close agreement with lithological information obtained from nearby boreholes.

### Engineering and geotechnical parameters

This section details the secondary geotechnical indicators derived from primary seismic parameters compressional wave velocity (Vp), shear wave velocity (Vs), and bulk density (ρ). These derived values provide a quantitative framework for assessing material stiffness, compressibility, and load-bearing performance, which are essential for evaluating foundation stability and seismic hazard potential^38^.

#### Dynamic elastic moduli

Elastic moduli describe how subsurface materials respond to stress and strain. These constants offer complementary insights into soil and rock behavior under both static and dynamic loading conditions^39^.

The most applied elastic constants in geotechnical and seismic studies include Young’s modulus, shear modulus, bulk modulus, Poisson’s ratio, and Lame’s parameters. Each provides complementary insights into soil and rock behavior under static and dynamic loading conditions^21^.

Firstly, we will use a Gardener equation to calculate the density to use it for elastic moduli^40^:

1 $$\:\rho\:=\:a{V}_{p}^{0.25}$$

V_p_ in m/s, a = 0.31 when the density is given in g/cm^3^.

This empirical relationship was calibrated using wireline logging data from Suez well-3 to ensure precise density assignments for the region’s specific lithological units.

##### Young’s modulus (E)

Young’s modulus Measures axial stiffness and is used to estimate settlement behavior under foundation loads. High values indicate dense, lithified materials^41–43^.

2 $$\:E=\rho\:{V}_{S}^{2}\:\left(\frac{3{V}_{P}^{2}-4{V}_{S}^{2}}{{V}_{P}^{2}-{V}_{S}^{2}}\right)$$

where: V_p_ and V_s_ are primary and shear wave velocities, $\:\rho\:\:$is rock density.

##### Shear modulus (G)

The shear modulus, also known as the rigidity modulus, it quantifies resistance to shear stress and reflects the inherent strength of the soil skeleton or rock framework^44^.

3 $$\:G=\rho\:{V}_{S}^{2}$$

where: V_s_ are shear wave velocities, $\:\rho\:\:$is rock density.

##### Bulk modulus (K)

The bulk modulus measures resistance to uniform compression, representing how a material responds to changes in volume under hydrostatic pressure. A low bulk modulus reflects highly compressible soils, while higher values characterize more consolidated or cemented materials with lower compressibility^45,46^.

4 $$\:K=\rho\:({V}_{P}^{2}-\frac{3}{4}{V}_{S}^{2})$$

where: V_p_ and V_s_ are primary and shear wave velocities, $\:\rho\:\:$is rock density.

##### Poisson’s ratio (σ)

Poisson’s ratio relates lateral strain to longitudinal strain under axial loading. It is highly sensitive to lithology, porosity, degree of saturation, and clay content. Values approaching **0.5** signify saturated or weakly consolidated materials, while very low or near-zero values indicate strong, brittle, or highly competent rocks. Negative values, though rare, may occur in anisotropic or highly fractured formations^47,48^.

5 $$\:\sigma\:=\frac{{({V}_{P}/{V}_{S})}^{2}-2}{{2({V}_{P}/{V}_{S})}^{2}-2}$$

where: σ is Poisson Ration, V_p_ and V_s_ are primary and shear wave velocities.

##### Lame’s parameter (λ)

Lame’s parameter (λ), in conjunction with the shear modulus, is used in elasticity theory to describe stress-strain relationships in isotropic materials. This parameter is important in advanced geophysical modeling, particularly for seismic wave propagation studies and rock physics applications^48,49^.

6 $$\:{\uplambda\:}=\frac{\sigma\:E}{(1+\sigma\:)(1-2\sigma\:)}$$

where: σ is Poisson Ration.

Integrating these moduli allows for the identification of weak zones and the delineation of soil-rock interfaces.

#### Applied engineering indices and performance parameters

These parameters bridge the gap between geophysical imaging and geotechnical design by serving as proxies for subsurface competence.

##### Material competence indices

*Stress Ratio (S*_*i*_ *).* The stress ratio expresses the balance between vertical and lateral strain in soils under load. Larger values are generally associated with loose, water-rich, or fine-grained soils, whereas smaller values indicate denser and more competent ground^50,51^.

7 $$\:{S}_{i}=\frac{\sigma\:}{1-\sigma\:}$$

where: σ is Poisson’s Ratio.

*Concentration Index (C*_*i*_ *)*. C_i_ is a velocity-ratio based competence parameter often used to assess the degree of material consolidation. Higher values indicate stiffer and more competent deposits, while lower values mark weak or compressible horizons^50,52^.

8 $$\:{C}_{i}=\frac{1+\sigma\:}{\sigma\:}$$

where: σ is Poisson’s Ratio.

*Material Index (V)*. V is a qualitative measure of the “material quality” in relation to foundation stability, derived from elastic constants and velocity ratios. Higher V values generally correspond to stronger, more compact materials, while negative or low values indicate weak or fractured deposits^42,53^.

9 $$\:V=\frac{G-{\uplambda\:}}{G+{\uplambda\:}}=\left(1-4\sigma\:\right)$$

where: λ is Lame’s Constant. G is the Shear Modulus, σ is Poisson’s Ratio.

##### Foundation performance and stability

The estimated ultimate bearing capacity (qult) and settlement (δ) values were derived from dynamic elastic moduli obtained from seismic velocity profiles and should therefore be regarded as preliminary geotechnical indicators rather than design parameters. These results provide a reliable, spatially continuous assessment of subsurface stiffness and competence, supporting site screening and geohazard evaluation. However, they are intended to complement not replace site-specific geotechnical investigations, including borehole data and static load testing, which remain essential for final foundation design.

*Ultimate Bearing Capacity (q*_*ult*_ *)*. The maximum contact stress that the soil or rock can sustain before failure. Bearing capacity increases with density and shear-wave velocity, reflecting improved foundation competence^41,54^.

10 $$\:{q}_{ult}=1/100\rho\:{V}_{S}$$

where: Vs is shear wave Velocity, $\:\rho\:\:$is rock density.

*Settlement (δ)*. Settlement is the downward deformation of soil under structural loads, predominantly elastic at small strains. Larger moduli correspond to smaller settlements, whereas soft or compressible deposits yield higher values^41,55^.

11 $$\:{\delta\:}_{z}=\frac{{q}_{ult}}{E}Z$$

12 $$\:{Z}^{2}=\frac{3}{4\pi\:}\frac{{q}_{ult}}{0.333}$$

where: q_ult_ for the load at unit area is the stress value depending on the depth z, δ_z_ is the settlement value for the soil column with the depth z.

*Standard Penetration Resistance (N-value)*. The Standard Penetration Test (SPT) blow count (N) is a primary index for evaluating the density of cohesionless soils and their liquefaction potential the tendency of saturated soils to lose strength under cyclic loading. In this study, N-values are estimated for the surficial unconsolidated wadi sediments (Layer 1) using the velocity-based correlation^56–58^.

13 $$\:{V}_{s}=89.9*{N}^{0.341}$$

where: Vs is shear wave Velocity.

This empirical relation is specifically calibrated for shallow, cohesionless materials where low Vs and N-values indicate higher liquefaction susceptibility. For the deeper, competent sandstone bedrock, N-values are reported as ‘equivalent resistance’ to provide a consistent scale of material stiffness, although liquefaction terminology is not applicable to these lithified units.

#### Vs30

The average shear-wave velocity to a depth of 30 m ($\:{V}_{s30}$) is the standard metric for seismic hazard assessment. It is calculated by accounting for the thickness and velocity of each individual layer within the top 30-meter interval^25^.

14 $$\:{V}_{s30}=\frac{30}{\sum\:_{i=1}^{N}\left(\frac{{d}_{i}}{{V}_{si}}\right)}$$

where $\:{d}_{i}$ is the thickness (in meters) of the ith soil/rock layer within the top 30 m, and Vsi is the corresponding shear-wave velocity (in m/s). This formulation accounts for the contribution of each layer to the total travel time of shear waves over the depth interval.

This index is used to categorize the area into NEHRP Site Classes (A through E), ranging from hard rock to soft soil  (Table 2). These classifications directly determine the ground-motion amplification factors and design spectra required for safe engineering in seismic codes^59,60^.

**Table 2:** Summarizes the standard site classes according to NEHRP (BSSC, 2004). These classes relate Vs30 ranges to site conditions and expected ground motion amplification.

Site Class	Vs30 Range (m/s)	General Description	Typical Materials
A	> 1500	Hard rock	Fresh, intact igneous and metamorphic rocks
B	760–1500	Rock	Competent rock or very stiff formations
C	360–760	Very dense soil/soft rock	Dense sand, gravel, stiff clay, weakly cemented rock
D	180–360	Stiff soil	Medium dense sand, sandy clay, firm deposits
E	< 180	Soft soil	Loose alluvial deposits, peat, soft clays, highly weathered soils

#### Uncertainty analysis and error propagation

The geotechnical parameters derived in this study are computed from P-wave velocity, S-wave velocity, and bulk density, each of which carries measurement uncertainty inherited from their respective tomographic and MASW inversions. Based on the uncertainty analysis and error propagation parameters, the mean relative uncertainties for the seismic and derived geotechnical properties can be summarized in Table 3.

**Table 3:** Mean relative uncertainties for seismic and dynamic geotechnical properties.

Parameter	Symbol	Mean Relative Uncertainty (%)	Parameter	Symbol	Mean Relative Uncertainty (%)
P-wave Velocity	V_p_	3.89%	Shear Modulus	G	9.08%
S-wave Velocity	V_s_	4.51%	Young’s Modulus	E	13.33%
Bulk Density	ρ	0.97%	Poisson’s Ratio	$\:\sigma\:$	1.79%
Time-averaged Shear-wave Velocity (30 m)	V_s_30	5.42%	Ultimate Bearing Capacity	q_ult_	4.61%

Uncertainty and reliability analyses demonstrated that the MASW inversion produced robust and consistent subsurface models. Constrained by corresponding P-wave velocity (Vp) models to minimize inversion non-uniqueness, the inversion achieved an average dispersion-curve RMS misfit of 6.36 ± 1.26%, with a phase-velocity uncertainty of 28.8 ± 8.5 m/s. The mean relative uncertainty of the derived S-wave velocity (Vs) models across the eighteen profiles was 4.51%, indicating high model stability. The reliability of the inversion results was further validated through strong agreement with independent lithological and wireline log data from Suez Well-3, confirming the identified three-layer subsurface structure. Although the inversion results remain influenced by the assumed initial Vp/Vs ratios (1.65–1.90) and the inherent trade-off between resolution and the maximum investigation depth (~ 40 m), the derived Vs profiles provide high-confidence estimates within the upper 30 m, ensuring reliable Vs30 determination and NEHRP site classification.

## Results and discussions

The interpretation of eighteen P-wave and S-wave seismic refraction profiles characterized the shallow subsurface, delineating major lithological units, while also capturing structural features associated with the tectonic evolution of the Gulf of Suez rift system. Integration with borehole and wireline data enhanced interpretation reliability (Table 4).

**Table 4:** The velocity parameters for the first, second, and third layers and their RMS error, together with the depths to the tops of the second and third layers, were determined for each of the 18 seismic refraction profiles.

Profile No.	First Layer		Second Layer		Third Layer		V_*p*_	V_s_	Depth to the Top of	
	V_*p*_ (m/s)	V_s_ (m/s)	V_*p*_ (m/s)	V_s_ (m/s)	V_*p*_ (m/s)	V_s_ (m/s)	RMS (%)	RMS (%)	Second Layer	Third Layer
1	527	296	1565	777	2780	1354	3.4	6	−9.2	−19.7
2	835	464	2078	1071	3076	1789	4.1	8.3	−6.3	−14.5
3	659	355	2056	1076	2954	1658	3.7	7.4	−4.2	−18.9
4	1277	691	2145	1186	3200	1609	4.5	6.9	−3.1	−14.7
5	536	295	1659	861	3515	1660	3.2	5.1	−4.7	−15.1
6	748	428	1902	1088	3000	1711	4.8	5.1	−2.5	−21.2
7	715	383.	1909	1087	3116	1597	3.9	4.7	−4.8	−14.7
8	682	342	1555	910	2297	1168	4.3	8	−4.9	−19.2
9	698	38	1860	936	2549	1443	3.1	6.9	−1.6	−22.7
10	885	459	1833	1032	3035	1705	3.6	7.3	−3.9	−21.1
11	653	343	1819	899	2951	1478	4.7	4.6	−1.6	−22.4
12	634	367	1807	1028	2978	1484	3.3	8.4	−2.6	−24.9
13	600	330	1746	1026	3183	1768	4	7.8	−2.5	−22.2
14	610	353	1457	713	2668	1395	4.9	5.3	−5.1	−21.6
15	500	270	1431	769	2264	1134	3.5	5.2	−3.6	−21.1
16	880	474	1838	1057	2790	1616	4.2	5.2	−2.8	−23.2
17	884	493	1896	1062	3027	1563	3	5.7	−1.4	−19.3
18	637	377	2029	1072	3014	1630	4.6	6.6	−5.6	−24.1

The seismic models consistently resolve three primary geo-seismic layers, each with characteristic velocity ranges, lithological associations, and thickness variations that are critical for geotechnical assessment (Fig. 6).

- First Layer: Unconsolidated Wadi Sediments.

The surficial unit is composed of unconsolidated wadi deposits characterized by low seismic velocities. P-wave values (V_p1_) range between 400 and 1100 m/s, while shear-wave velocities (V_s1_) range from approximately 130 to 550 m/s. This layer is relatively thin (0.5–10 m) and represents poorly compacted, heterogeneous surface materials that are prone to settlement and deformation under applied loads.

- Second Layer: Consolidated Wadi Sediments.

Overlying the bedrock is a zone of consolidated wadi sediments with moderately higher velocities. P-wave velocities (V_p2_) vary from 1200 to 2000 m/s, and corresponding shear-wave velocities (V_s2_) are 545–1100 m/s. The thickness of this unit ranges from 8.5 to 25 m. These values indicate partial cementation, most likely calcareous, which enhances stiffness and reduces compressibility relative to the upper layer.

- Third Layer: Sandstone Bedrock.

The basal horizon corresponds to a competent sandstone formation forming the load-bearing bedrock. This unit exhibits high P-wave velocities of 2200–3500 m/s and shear-wave velocities range from 1200 to 1800 m/s. It constitutes the primary foundation-bearing layer and provides the highest stiffness and strength for engineering applications.

**Fig. 7:**
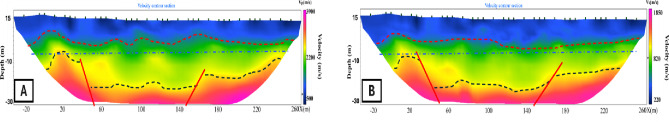
Example of tomographic inversion results for seismic refraction Profile 1. (**A**) P-wave derived from SRT model. (**B**) S-wave derived 2D velocity model. The tomograms reveal three distinct geoseismic layers; the upper boundary marks the top of the sandstone bedrock, while the red dashed line indicates the base of the unconsolidated wadi sediments. Solid red lines denote the interpreted fault structure. Blue dashed line indicates the static groundwater level from Suez Well-3.F.

The lateral variability within these layers reflects both lithological heterogeneity and the influence of extensional tectonics. Several tomograms display abrupt velocity contrasts, thickness changes, and tilted layering consistent with fault offsets and half-graben development, features widely documented in the rifted margin of the Gulf of Suez. These structural discontinuities generate irregular bedrock topography, with the sedimentary cover locally thickening in downthrown blocks and thinning over horsts. Such irregularities must be accounted for in foundation design, as they directly influence differential settlement and the required depth of foundation elements.

To provide a more comprehensive visualization of subsurface heterogeneity, the 2D velocity tomograms were interpolated to produce a quasi-3D visualization (Fig. 7). This composite velocity field highlights both the continuity of the three major layers and their spatial variability across the survey area.

**Fig. 8:**
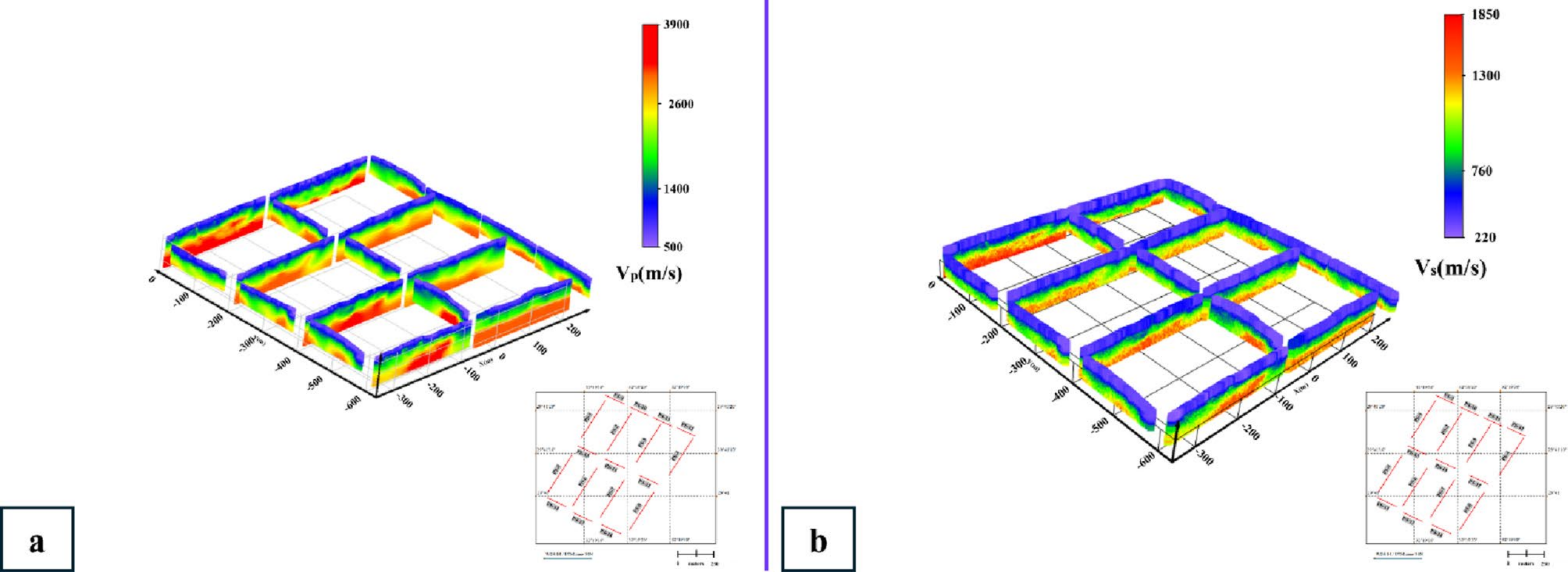
Quasi-3D visualization of the survey area, where (**a**) represents the 3D seismic visualization of the eighteen spreads for P-waves and (**b**) represents the eighteen spreads for S-waves.

The unconsolidated wadi sediments form a discontinuous veneer, locally thinning to 1 m and thickening to nearly 10 m. The consolidated wadi deposits beneath exhibit variable thicknesses of 8.5–25 m, reflecting both depositional and tectonic influences. The sandstone bedrock is consistently imaged as a high-velocity horizon, although its upper surface is highly irregular due to faulting and basement block rotation.

Several low-velocity anomalies are apparent within the quasi-3D visualization, interpreted as fault zones or fractured intervals. These appear as steep velocity gradients and discontinuities, consistent with graben- and half-graben geometries of the rift system. Such features represent structurally weak zones that may act as pathways for groundwater, amplify ground motion, or localize deformation during seismic events.

The seismic interpretation correlates strongly with borehole and well-log data from nearby sites. Wireline logs confirm the presence of calcareous-cemented sandstones beneath the unconsolidated cover, with resistivity and porosity values consistent with the seismic velocity assignments. Deeper sections correspond to compacted argillaceous sandstones forming the bedrock. This agreement underscores the reliability of combining P-wave and S-wave refraction methods for resolving both lithological and structural variations in the near surface.

A detailed assessment of the subsurface at Ain El Sokhna was carried out using compressional and shear wave velocity data together with derived density estimates. These parameters provide a robust framework for evaluating the stiffness, strength, and deformation properties of the encountered soils and rocks, which are critical for both geotechnical design and seismic response analysis.

**Fig. 9:**
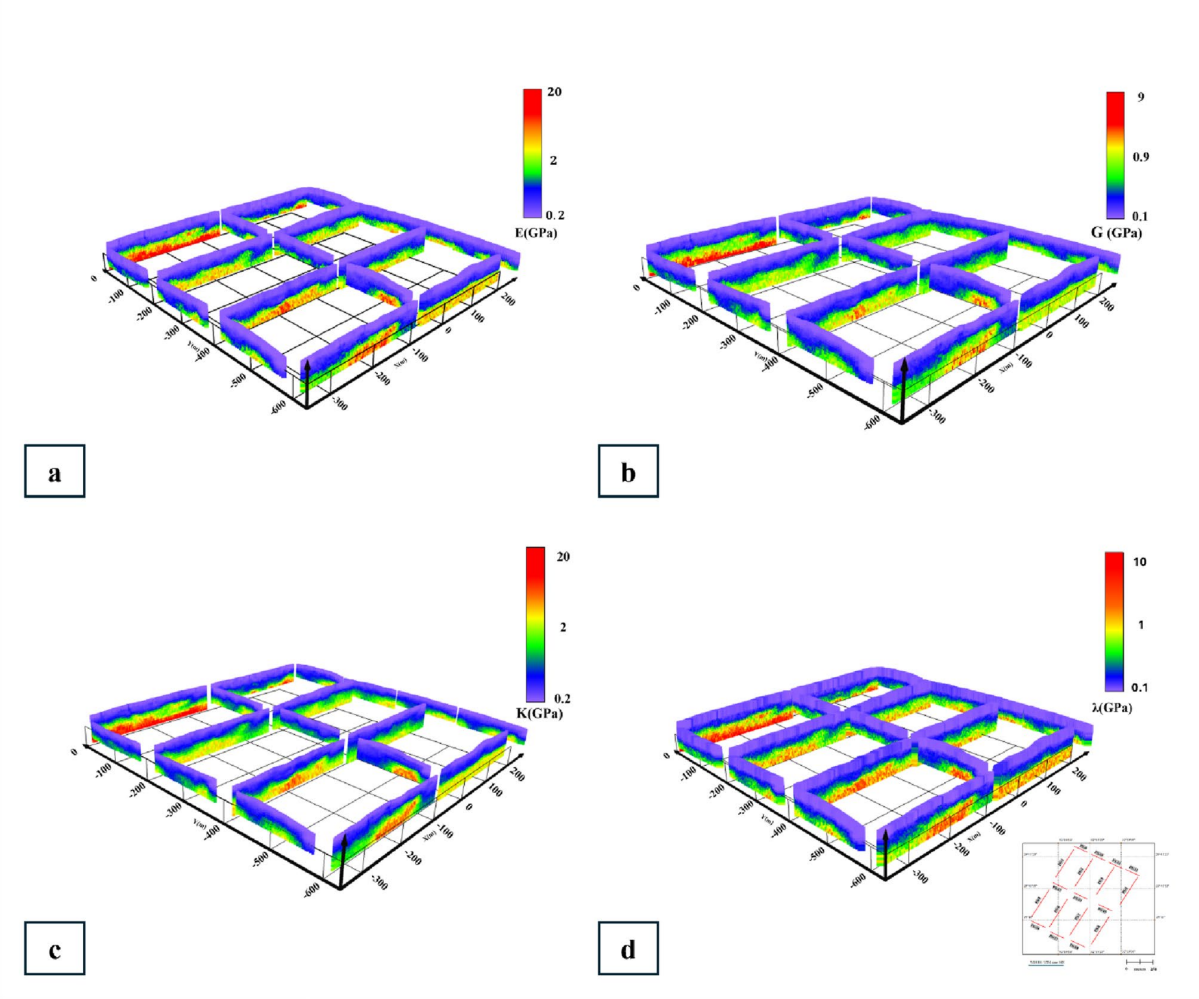
Quasi-3D visualization of the elastic moduli, showing (**a**) Young’s modulus, (**b**) shear modulus, (**c**) bulk modulus, and (**d**) Lame’s first parameter.

The bulk density increases systematically with depth, reflecting the combined influence of lithology and burial compaction. The near-surface wadi deposits show relatively low densities of 1350–1550 kg/m³, consistent with their unconsolidated nature. The underlying semi-cemented wadi sediments exhibit higher densities, between 1770 and 1860 kg/m³, while the sandstone bedrock is the densest unit, with values of 2060–2400 kg/m³. This progression follows Gardner’s velocity density relationship, confirming the improved competence of deeper units.

Poisson’s ratio illustrates a similar depth-dependent trend. The shallow deposits exhibit high values characteristic of poorly consolidated, heterogeneous soils. The middle horizon yields values from 0.34 to 0.31, suggesting partially compacted materials, while the bedrock shows low ratios between 0.28 and 0.21, consistent with moderately stiff rocks.

The Four principal elastic moduli shear modulus (G), Young’s modulus (E), bulk modulus (K), and Lame’s Parameter (λ) all show increasing magnitudes with depth. (Fig. 8)

In the wadi deposits, G varies between 0.069 and 0.381 GPa, E between 0.043 and 0.89 GPa, K between 0.046 and 0.91 GPa, and λ between 0.074 and 0.63 GPa, indicating weak, compressible material.

For the semi-cemented sediments, values increase to G varies between 0.74 and 2.1 GPa, E between 0.94 and 6.3 GPa, K between 0.46 and 6.1 GPa, and λ between 0.82 and 3.3 GPa, pointing to enhanced stiffness.

The sandstone bedrock demonstrates the highest values (G = 2.2–7.91 GPa, E = 6.78–14.2 GPa, K = 7.30–45 GPa, λ = 3.42–10.7 GPa), consistent with strong, competent rock (Table 5).

**Table 5:** Summary of the calculated density, Dynamic Elastic Moduli, Vs₃₀ values, geotechnical parameters.

Profile no.	Layer	Dynamic Elastic Moduli and Density						Geotechnical Parameters				
		ρ (kg/m³)	σ	G (GPa)	E (GPa)	λ (GPa)	K (GPa)	Si	Ci	V	q_ult_ (Pa)	δ (m)
1	1	1486	0.369	0.13	0.331	0.152	0.239	0.568	3.714	−0.177	13.053	9.01 × 10^− 4^
	2	1950	0.336	1.176	3.15	2.42	3.20	0.507	3.973	−0.345	117.698	2.57 × 10^− 3^
	3	2251	0.244	4.131	11.1	9.14	1.19	0.335	4.904	0.177	413.113	3.69 × 10^− 3^
2	1	1666	0.377	0.358	0.915	0.445	0.684	0.583	3.608	−0.109	35.805	1.34 × 10^− 3^
	2	2093	0.319	2.4011	6.33	4.23	5.84	0.469	4.134	−0.276	240.120	2.80 × 10^− 3^
	3	2309	0.244	7.387	18.4	7.06	12	0.323	5.091	0.122	738.740	4.31 × 10^− 3^
3	1	1571	0.396	0.197	0.512	0.288	0.419	0.521	3.740	−0.186	19.753	1.10 × 10^− 3^
	2	2087	0.311	2.415	6.33	3.99	5.6	0.452	4.211	−0.246	241.517	2.78 × 10^− 3^
	3	2285	0.27	6.278	15.9	7.38	11.6	0.370	4.702	0.281	627.866	4.14 × 10^− 3^
4	1	1853	0.396	0.885	2.29	1.25	0.184	0.414	3.514	−0.172	88.504	2.03 × 10^− 3^
	2	2110	0.312	2.965	7.59	3.77	5.75	0.389	4.573	−0.119	296.582	3.00 × 10^− 3^
	3	2332	0.268	6.036	16.1	11.8	15.8	0.334	5.023	0.232	603.632	4.19 × 10^− 3^
5	1	1491	0.37	0.130	0.334	0.167	0.254	0.591	3.558	−0.124	13.032	9.05 × 10^− 3^
	2	1978	0.325	1.465	3.86	2.52	3.49	0.462	4.165	−0.264	146.521	2.67 × 10^− 3^
	3	2387	0.291	6.576	17.8	16.3	20.7	0.354	4.805	0.261	657.675	4.38 × 10^− 3^
6	1	1621	0.378	0.297	0.747	0.313	0.511	0.545	3.903	−0.025	29.733	1.25 × 10^− 3^
	2	2047	0.333	2.425	6.10	2.56	4.18	0.445	4.296	−0.027	242.528	2.74 × 10^− 3^
	3	2294	0.256	6.721	16.9	7.20	11.7	0.349	4.866	0.235	672.113	4.19 × 10^− 3^
7	1	1603	0.373	0.235	0.611	0.348	0.505	0.525	3.530	−0.193	23.527	1.16 × 10^− 3^
	2	2049	0.333	2.420	6.10	2.63	4.24	0.452	4.843	−0.041	242.059	2.74 × 10^− 3^
	3	2316	0.212	5.905	15.6	10.7	14.6	0.375	5.105	0.288	590.512	4.14 × 10^− 3^
8	1	1584	0.325	0.185	0.493	0.365	0.489	0.497	4.014	−0.327	18.516	1.10 × 10^− 3^
	2	1947	0.278	1.610	3.99	1.49	2.56	0.416	4.165	0.040	161.055	2.65 × 10^− 3^
	3	2146	0.247	2.929	7.77	5.46	7.41	0.382	5.073	0.302	292.945	3.32 × 10^− 3^
9	1	1593	0.309	0.233	0.599	0.309	0.465	0.599	3.506	−0.141	23.300	1.15 × 10^− 3^
	2	2036	0.283	1.783	4.75	3.47	4.66	0.493	4.027	−0.321	178.392	2.69 × 10^− 3^
	3	2203	0.248	4.583	11.6	5.14	8.19	0.359	4.784	0.057	458.323	3.67 × 10^− 3^
10	1	1691	0.377	0.356	0.937	0.612	0.849	0.562	3.650	−0.264	35.618	1.35 × 10^− 3^
	2	2028	0.32	2.160	5.48	2.49	3.93	0.466	4.732	−0.072	216.033	2.71 × 10^− 3^
	3	2301	0.221	6.694	17.1	7.81	12.3	0.368	4.915	0.177	669.438	4.21 × 10^− 3^
11	1	1567	0.334	0.184	0.482	0.299	0.422	0.548	4.231	−0.238	18.405	1.08 × 10^− 3^
	2	2025	0.321	1.635	4.38	3.43	4.52	0.512	4.953	−0.354	163.529	2.62 × 10^− 3^
	3	2285	0.228	4.994	13.3	9.91	13.2	0.398	5.008	0.330	499.430	3.84 × 10^− 3^
12	1	1555	0.389	0.209	0.523	2.05	3.45	0.528	3.520	−0.013	20.993	1.12 × 10^− 3^
	2	2021	0.331	2.135	5.38	2.33	3.75	0.453	4.370	−0.043	213.525	2.70 × 10^− 3^
	3	2290	0.219	5.043	13.5	10.2	13.6	0.353	4.987	0.339	504.339	3.86 × 10^− 3^
13	1	1534	0.373	0.166	0.428	0.219	0.330	0.597	3.522	−0.136	16.667	1.00 × 10^− 3^
	2	2004	0.345	2.108	5.21	1.89	3.30	0.410	4.226	−0.540	210.841	2.68 × 10^− 3^
	3	2328	0.247	7.275	18.6	9.04	13.9	0.383	5.100	0.108	727.561	4.35 × 10^− 3^
14	1	1541	0.387	0.192	0.479	0.189	0.317	0.530	4.026	−0.006	19.197	1.08 × 10^− 3^
	2	1915	0.323	2.745	2.62	2.12	2.77	0.481	4.200	−0.370	97.455	2.44 × 10^− 3^
	3	2228	0.232	4.337	11.4	7.18	10.1	0.353	5.209	0.247	433.754	3.67 × 10^− 3^
15	1	1466	0.393	0.107	0.277	0.152	0.224	0.515	3.411	−0.173	10.723	8.35 × 10^− 3^
	2	1907	0.32	1.128	2.93	1.65	2.40	0.422	4.369	−0.187	112.880	2.50 × 10^− 3^
	3	2138	0.225	2.749	7.33	5.47	7.30	0.398	5.006	0.331	274.961	3.29 × 10^− 3^
16	1	1688	0.376	0.381	0.985	0.547	0.801	0.519	3.890	−0.180	38.023	1.38 × 10^− 3^
	2	2030	0.323	2.265	5.68	2.33	3.84	0.439	4.460	−0.014	226.592	2.72 × 10^− 3^
	3	2253	0.238	5.886	14.7	5.77	9.69	0.329	5.042	0.100	588.627	3.97 × 10^− 3^
17	1	1690	0.375	0.412	1.05	0.497	0.771	0.576	3.658	−0.094	41.171	1.41 × 10^− 3^
	2	2045	0.325	2.308	5.87	2.73	4.27	0.472	4.689	−0.084	230.831	2.73 × 10^− 3^
	3	2299	0.244	5.617	14.8	9.83	13.6	0.367	5.142	0.273	561.731	4.04 × 10^− 3^
18	1	1557	0.391	0.222	0.545	0.188	0.335	0.528	3.610	−0.083	22.160	1.13 × 10^− 3^
	2	2081	0.322	2.395	6.26	3.78	5.38	0.441	4.268	−0.224	239.532	2.77 × 10^− 3^
	3	2297	0.232	6.105	15.8	8.65	12.7	0.345	5.110	0.173	610.522	4.14 × 10^− 3^

Engineering parameters were derived from seismic velocities, elastic moduli, and densities obtained from SSRT bedrock interpretation. The full results are mapped in Fig. 9. Examination of these outputs highlights several key observations:

The derived geotechnical indices for the sandstone bedrock display consistently higher competence compared to the overlying sediments. Stress ratio (Si) values for the bedrock are the lowest of the investigated sequence, varying between 0.33 and 0.39, which is characteristic of compacted, well-consolidated material. A gradual increase in Si is observed toward the eastern part of the study area.

The concentration index (Ci) ranges from 4.74 to 5.22, reflecting compacted conditions, with the highest values concentrated in the central and southern sectors. The material index (V) of the bedrock spans 0.05 to 0.35, with elevated values occurring in the eastern portion of the site, consistent with more competent zones.

**Table 6:** Summary of representative Standard Penetration Resistance (N-value) ranges and their corresponding geotechnical interpretations.

Layer	*N*-value	Interpretation
Layer 1	5–22	Low values indicating loose, poorly compacted surface materials
Layer 2	23–45	Reflecting the transition to semi-cemented materials with improved stiffness
Layer 3	30–80	Signifying very stiff, well-consolidated bedrock capable of supporting heavy loads

The ultimate bearing capacity (qult) of the bedrock is substantial, ranging from 80 to 360 Pa, with the strongest capacities identified in the north-western areas and diminishing toward the south-east. Settlement (δ) estimates are minimal, ranging between 3.0 × 10⁻³ and 4.5 × 10⁻³ m, with the smallest values located in the south-eastern direction. Finally, the Standard Penetration Resistance (N-value) lies between 30 and 80, with the highest susceptibility indicated in the north-western part of the surveyed region.

**Fig. 10:**
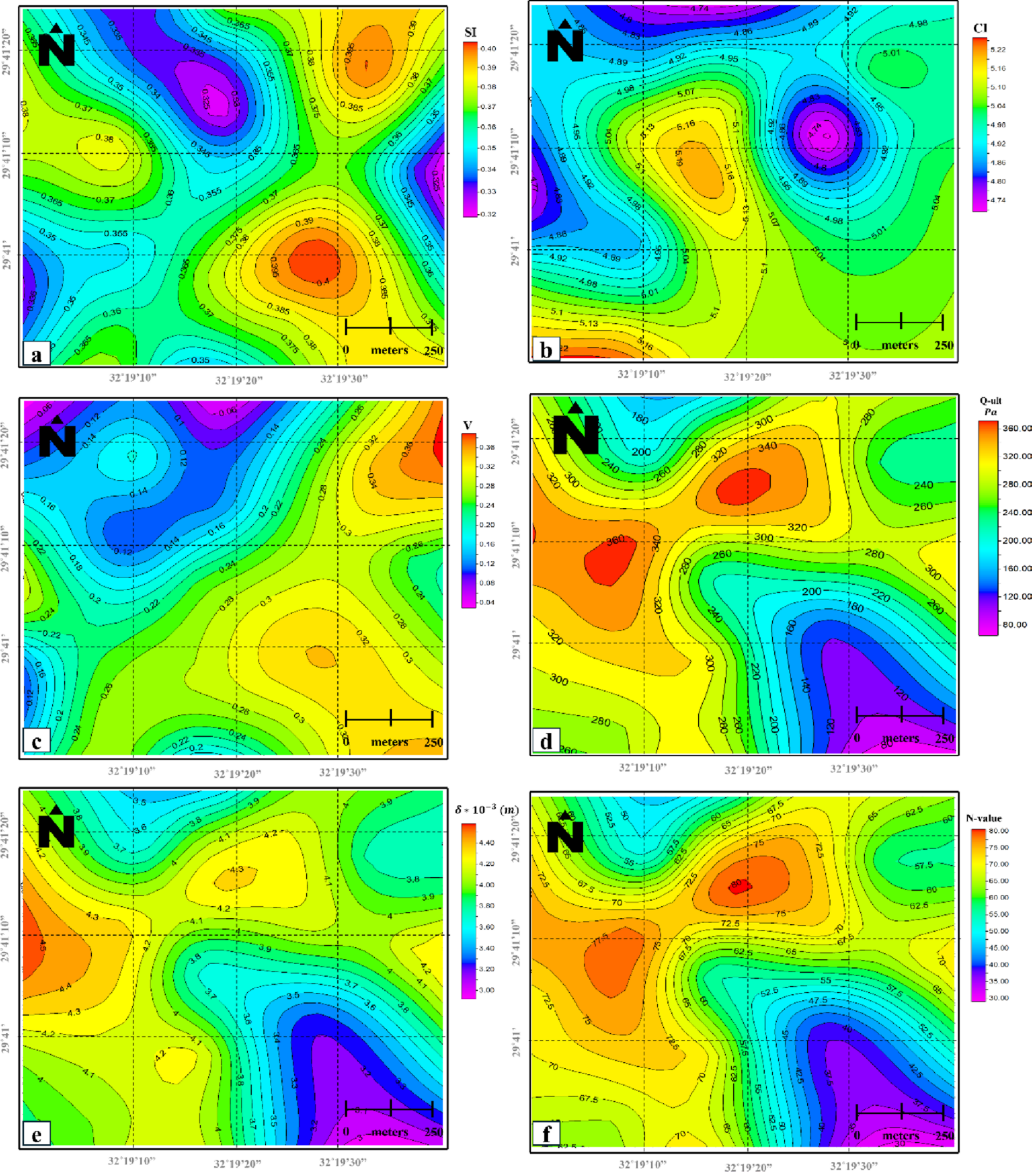
2D maps illustrate the lateral distribution of the bedrock’s engineering parameters: (**a**) Stress Ratio, (**b**) Concentration Index, (**c**) Material Index, (**d**) Ultimate Bearing Capacity, (**e**) Settlement, and (**f**) Bedrock Competence (Equivalent N-value).

By calculating the Vs₃₀ for each model the survey provides a spatially comprehensive assessment of the site’s dynamic response. The resulting values for all 18 profiles range from approximately 763 m/s to 1261 m/s (Table 6), consistently placing the entire investigated area within NEHRP Site Class B (Table 7).

**Table 7:** Summary of the calculated Vs₃₀ values and NEHRP site class for the investigated area.

Profile No.	Vs30 (m/s)	NEHRP site class	Profile No.	Vs30 (m/s)	NEHRP site class
Profile 1	775.2	B	***Profile 10***	987.4	B
Profile 2	1002.9	B	***Profile 11***	910.3	B
Profile 3	931.4	B	***Profile 12***	931.4	B
Profile 4	1261.6	B	***Profile 13***	961.4	B
Profile 5	811.6	B	***Profile 14***	788.1	B
Profile 6	1065.3	B	***Profile 15***	783.3	B
Profile 7	960.9	B	***Profile 16***	1019.8	B
Profile 8	763.4	B	***Profile 17***	1130.6	B
Profile 9	943.8	B	***Profile 18***	840.2	B

A detailed evaluation of the computed geotechnical parameters indicates that the sandstone bedrock generally exhibits fair to good competence, providing a reliable foundation horizon (Fig. 10). However, the 2D tomograms and quasi-3D visualizations reveal localized zones of reduced competence interpreted as faulted or fractured intervals which introduce spatial variability in stiffness and load-bearing depth. Despite these structural heterogeneities, the time-averaged shear-wave velocity for the top 30 m (Vs₃₀) consistently remains above the 760 m/s threshold across all 18 profiles. Consequently, the site is classified as NEHRP Site Class B, confirming that while localized anomalies exist, the overall subsurface is dominated by competent rock suitable for heavy industrial infrastructure.

## Discussion

This study demonstrates that the joint application of P-wave and S-wave tomography provides a reliable and effective framework for geotechnical site characterization in the tectonically active Ain Sokhna area. Inversion of first-arrival travel times produced high-resolution velocity tomograms that clearly resolved three principal subsurface units. The uppermost layer consists of unconsolidated alluvial (wadi) sediments, characterized by low seismic velocities (V_p_ = 400–1100 m/s; V_s_ = 130–550 m/s) and poor mechanical competence. This is underlain by an intermediate unit of semi-consolidated wadi deposits exhibiting moderate velocities (V_p_ = 1200–2000 m/s; V_s_ = 545–1100 m/s), reflecting partial cementation and improved stiffness. The deepest unit corresponds to competent sandstone bedrock, defined by high velocities (V_p_ = 2200–3500 m/s; V_s_ = 1200–1800 m/s), which constitutes the primary load-bearing horizon. Consistent with the velocity structure, the derived elastic parameters show a systematic increase in stiffness with depth, from low shear moduli in the near-surface soils (G on the order of 0.01–0.1 GPa) to markedly higher rigidity within the bedrock (G ≈ 2–7.9 GPa; E ≈ 6.8–14.2 GPa). The calculated Vs₃₀ values for all profiles correspond to NEHRP Site Class B, indicating generally favorable seismic site conditions associated with rock or very stiff ground.

Notwithstanding the overall competence of the subsurface, the tomographic results and quasi-3D visualizations reveal pronounced lateral heterogeneity within the sandstone bedrock. Several localized low-velocity anomalies, marked by steep velocity gradients and lateral discontinuities in both V_p_ and V_s_, are interpreted as faulted or fractured zones. These features are consistent with the NW-SE and NE-SW trending normal fault systems that accommodate extensional deformation within the Gulf of Suez rift. Their presence results in an irregular bedrock morphology, with thickening of the sedimentary cover in downthrown blocks and thinning over structural heights. From a geotechnical perspective, these zones represent localized reductions in stiffness and increases in compressibility relative to the surrounding intact bedrock. They may also influence local seismic response by concentrating deformation or amplifying ground motion, and could act as preferential pathways for groundwater flow, potentially enhancing weathering processes and further modifying mechanical properties.

The engineering parameters derived from the seismic data largely confirm the favorable bearing characteristics of the intact sandstone bedrock. This unit is characterized by low stress ratios (Si = 0.33–0.39), high concentration indices (C_i_ = 4.7–5.2), and positive material indices (V up to 0.3), all indicative of well-consolidated, stiff material. Correspondingly, the estimated ultimate bearing capacities (q_ult_ ≈ 80–360, in the adopted units) are high, while calculated settlements (δ ≈ 3 × 10^− 3^ −4.5 × 10^− 3^ m) remain minimal even under substantial loading. In contrast, the near-surface sediments are more compressible and are expected to contribute most significantly to total settlement. However, the occurrence of structurally weak, low-velocity zones within the bedrock introduces local variability in stiffness and load-bearing depth, increasing the potential for differential settlement. Consequently, although the site is generally underlain by competent rock, the results underscore the need for careful consideration of fault-related heterogeneities through targeted site-specific investigations and foundation designs that accommodate spatial variations in subsurface conditions, thereby ensuring the long-term safety and resilience of engineered structures in this active tectonic environment.

## Conclusions

The joint interpretation of P-wave and S-wave seismic refraction data, integrated with derived geotechnical parameters, provided a robust characterization of subsurface conditions at the Ain Sokhna site. The results delineate a generally competent sandstone bedrock with high stiffness, low compressibility, and Vs₃₀ values corresponding to NEHRP Site Class B, indicating favorable conditions for supporting heavy infrastructure. Nevertheless, localized low-velocity anomalies within the bedrock, interpreted as faulted or fractured zones, introduce lateral variability in stiffness and load-bearing depth that may influence seismic response and foundation performance. Accordingly, while the overall site conditions are suitable for development, these heterogeneities must be carefully accounted for in site-specific foundation design. This study underscores the value of integrated seismic tomography and geotechnical analysis for informed engineering decision-making in tectonically complex regions.

## Data Availability

Data will be made available on request.
